# Short-Term Insulin Requirements Following Gastric Bypass Surgery in Severely Obese Women with Type 1 Diabetes

**DOI:** 10.1007/s11695-014-1228-8

**Published:** 2014-03-26

**Authors:** Roeland J. W. Middelbeek, Tamarra James-Todd, Mary-Elizabeth Patti, Florence M. Brown

**Affiliations:** 1 Division of Endocrinology, Diabetes and Metabolism, Beth Israel Deaconess Medical Center, 330 Brookline Ave, Boston, MA USA; 2 Division of Women’s Health, Brigham and Women’s Hospital, 1620 Tremont St., 3rd floor, Boston, MA 02120 USA; 3 Joslin Diabetes Center, One Joslin Place, Boston, MA 02215 USA; 4 Harvard Medical School, Boston, MA USA

**Keywords:** Gastric bypass surgery, Type 1 diabetes, Insulin requirements

## Abstract

**Background:**

In severely obese type 2 diabetes patients, gastric bypass surgery (GB) reduces body mass index (BMI) and hemoglobin A1c (HbA1c) and allows reduced doses of insulin and other medications. Data regarding the effects of GB on severely obese patients with type 1 diabetes are limited.

**Methods:**

Severely obese women with type 1 diabetes (*n* = 9) were studied immediately before and after GB (7.7 ± 5.8 weeks, mean ± SD).

**Results:**

On average, GB reduced mean BMI by 11 % and mean HbA1c by 0.9 % (from 8.0 to 7.1 %), with a parallel 38 % decrease in basal insulin requirements (expressed per kilogram of body weight).

**Conclusion:**

GB rapidly decreased BMI, HbA1c, and insulin requirements in severely obese women with type 1 diabetes. However, physiologic insulin replacement remains necessary in patients with type 1 diabetes.

## Background

Recent data demonstrate that gastric bypass surgery (GB) is a highly effective strategy for the treatment of type 2 diabetes. GB achieves long-term weight loss, decreases mortality [1], and rapidly normalizes hyperglycemia [2], permitting dosage reduction and/or withdrawal of diabetes medication. These effects are likely related to both the marked reduction in peripheral insulin resistance [3] and increased incretin and insulin secretion [4], which occur even prior to sustained weight loss [5–7].

By contrast, little is known about the effects of GB on severely obese patients with type 1 diabetes. Prior case reports showed mixed effects of GB on hemoglobin A1c (HbA1c) and insulin requirements in individuals with type 1 diabetes [8–11]. Given the limited studies in this increasingly obese population [12], we investigated the short-term effects of GB on weight, HbA1c, and insulin requirements in obese subjects with type 1 diabetes.

## Methods

### Study Population

The study was approved by the Institutional Review Board of the Joslin Diabetes Center, and informed consent was waived. The electronic medical record database of the Joslin Diabetes Center was queried for the terms “type 1 diabetes” and “gastric bypass” for visits which occurred between January 1, 2000 and April 1, 2012; a total of 27 individuals were found. Gastric bypass was defined as Roux-en-Y laparoscopic gastric bypass surgery. After excluding subjects without a date of surgery listed (14.8 %), not on insulin therapy (3.7 %), with missing pre- or postsurgery visit documentation, or a visit >20 weeks after surgery (48.1 %), nine individuals were included in the final cohort for analysis. Demographic data were collected from medical records.

Subjects were treated with either continuous subcutaneous insulin infusion (CSII) or multiple daily injections (MDI). For CSII-treated subjects, only the basal insulin contribution to the total daily dose of insulin was available for review. For MDI-treated subjects, basal and meal bolus insulin requirements were recorded. Medical records were reviewed in detail to confirm the diagnosis of type 1 diabetes based on clinical characteristics [13]. The diagnosis of type 1 diabetes was further supported by the presence of autoantibodies against glutamic acid decarboxylase (GAD), insulinoma-associated (IA-2), and insulin (IAA), and/or low or undetectable C-peptide levels in four out of four patients tested. Additionally, patients were diagnosed with type 1 diabetes at a mean age of 15 years (<12 years of age in 56 % of subjects).

### Data Analysis

Body mass index (BMI), HbA1c, and basal insulin doses were considered primary outcomes. Systolic and diastolic blood pressures were considered secondary outcomes. BMI was calculated as weight in kilogram/height in square meters. HbA1c values were collected from medical record data. Insulin requirements were expressed relative to body weight and presented as units per kilogram per day, and both absolute and relative differences between pre- and postoperative requirements were calculated.

Age, age at diagnosis, length of time with diabetes, height, weight, and blood pressure were considered as continuous variables, and mean and standard deviation were calculated. Treatment with auxiliary oral or injected diabetes medications was assessed. To determine whether our primary outcomes differed before and after GB, we conducted paired *t* tests and defined significance as *p* < 0.05. To compare the change in insulin dose with the change in weight, we used the Pearson correlation. Microsoft® Office Excel® 2007 was used for statistical analyses.

## Results

All subjects (*n* = 9) were female (mean age 40.3 ± 8.5 years) and had a mean duration of type 1 diabetes of 25.3 ± 8.9 years (range 17–46 years). The mean age at diagnosis was 15.0 ± 8.2 years (range 8–28 years) (Table 1). All subjects were treated with insulin; five patients used CSII, while four patients used MDI. Two subjects were also treated with metformin and pramlintide as adjunctive medications to promote weight loss [14]; both of these subjects had positive autoantibodies and low or undetectable C-peptide levels, confirming type 1 diabetes.

**Table 1:** Baseline characteristics and postsurgery changes

	Presurgery	Postsurgery	*p* values
Age at time of surgery (years)	40.3 ± 8.5		
Female sex—*N* (%)	9 (100)		
Height (cm)	167.8 ± 8.0		
Weight (kg)	122.2 ± 23.5		
Diabetes duration at time of surgery (years)	25.3 ± 8.9		
Mean age at DM diagnosis (years)	15.0 ± 8.2		
Low or undetectable C-peptide level—*N* (%)	4 (44 %)		
Autoantibodies present (GAD, IA2, IAA)—*N* (%)	3 (33 %)		
Insulin pump—*N* (%)	5 (56 %)		
Insulin pump and pramlintide	1 (11 %)		
Multiple daily injections—*N* (%)	4 (44 %)		
Multiple daily injections and metformin	1 (11 %)		
Supplemental medications—*N* (%)	2 (22 %)		
Pramlintide, metformin			
HbA1c (%)	8.0 ± 1.3	7.1 ± 0.9	<0.05
BMI (kg/m^2^)	43.8 ± 8.0	38.7 ± 8.6	<0.05
Basal insulin requirement (U/day)	59.7 ± 43.8	32.6 ± 20.9	<0.05
% Basal insulin compared to presurgery (*n* = 9)	N/A	54.6	
Basal insulin/kg/day (U/kg/day)	0.47 ± 0.28	0.29 ± 0.14	<0.05
% Basal insulin/kg/day compared to presurgery	N/A	61.8	
Meal bolus insulin requirement (U/day) (*n* = 4)	53.0 ± 27.3	18.1 ± 12.6	0.061
% bolus insulin compared to presurgery	N/A	40.1	
Meal bolus dose/kg/day (U/kg/day)	0.44 ± 0.24	0.15 ± 0.09	0.076
% bolus insulin/kg/day compared to presurgery	N/A	34.6	
Total daily insulin requirement (U/day) (*n* = 4)	113 ± 33.8	52.6 ± 28.2	<0.05
% TDD compared to presurgery	N/A	46.9	
Total daily dose/kg/day (U/kg/day)	0.93 ± 0.14	0.51 ± 0.26	<0.05
% TDD insulin/kg/day compared to presurgery	N/A	50.7	
Systolic blood pressure (mmHg)	124.0 ± 8.7	113.7 ± 22.0	0.14
Diastolic blood pressure (mmHg)	74.2 ± 7.4	67.3 ± 6.0	0.06
Supplemental medications—*N* (%)	2 (22 %)	0 (0 %)	
Assessment postsurgery (weeks)		7.7 ± 5.8	

Values are represented as mean ± SD. Baseline characteristics of type 1 diabetes subjects who underwent GB were obtained during the last presurgery visit. HbA1c, BMI, blood pressure, and insulin requirements were obtained during the first postsurgical visit (mean 7.7 ± 5.8 weeks) and the last presurgical visit

*TDD* total daily dose, *MDI* multiple dose injections

**Fig. 1:**
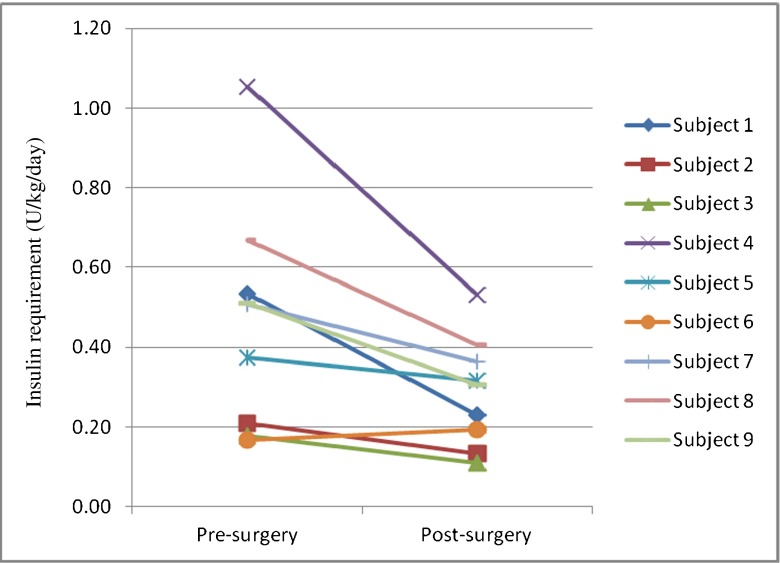
Basal insulin requirements per day (U/kg/day) from individual study participants (*n* = 9) were determined at the final presurgical and first postsurgical visits, between January 1, 2000 and April 1, 2012

At the time of the first postsurgical visit (mean 7.7 ± 5.8 weeks, range 2–18 weeks), BMI had decreased by a mean of 11 % (from 43.4 ± 8.0 to 38.7 ± 8.6 kg/m^2^, *p* < 0.05). HbA1c also decreased significantly, from 8.0 to 7.1 % (*p* < 0.05). The dose of basal insulin, calculated from recorded CSII and MDI regimens, decreased from 59.7 ± 43.8 to 32.6 ± 20.9 U/day (*p* < 0.05) (Table 1) and was also significantly decreased when expressed per kilogram of body weight (38 % reduction, from 0.47 ± 0.28 to 0.29 ± 0.14 U/kg/day, *p* < 0.05) (Fig. 1, Table 1). In MDI-treated subjects (*n* = 4), the meal bolus dose showed a trend towards a decrease by 65 % (from 0.44 ± 0.24 to 0.15 ± 0.9 U/kg/day, *p* < 0.08). Similarly, total daily insulin dose in MDI-treated patients, calculated from recorded basal and bolus regimens, decreased by a mean of 53 % from 113 ± 33.8 to 52.6 ± 28.2 U/day. Total daily insulin dose per kilogram of body weight also decreased by 49 % from 0.93 ± 0.14 to 0.49 ± 0.19 U/kg/day (*p* < 0.05). Subjects with postsurgical visits at <7.7 weeks showed a greater decrease in basal insulin requirement than those assessed at >7.7 weeks (−0.23 ± 0.19 vs. −0.11 ± 0.12 U/kg/day, respectively), but less weight loss (−10.2 ± 7.0 vs. −16.8 ± 10.1 kg, respectively). There was no statistically significant correlation between the extent of weight loss and percentage change in basal insulin requirements (Pearson correlation coefficient *r* = 0.34, *p* = 0.37).

None of the subjects was able to discontinue insulin therapy, consistent with their absolute insulin deficiency and type 1 diabetes, but all auxiliary oral and injectable therapy was discontinued after surgery. Both systolic and diastolic blood pressure tended to decrease, from 124 ± 9 to 114 ± 22 mmHg and from 74 ± 7 to 67 ± 6 mmHg (*p* = 0.14 and *p* = 0.06, respectively).

## Discussion

This study demonstrates that GB rapidly and significantly reduces BMI, HbA1c, and insulin requirements in severely obese women with type 1 diabetes. These patterns are similar to those in prior studies of patients with type 2 diabetes [15]. However, the 38 % reduction in basal insulin requirements in both CSII and MDI-treated patients with type 1 diabetes and 50 % reduction in total insulin requirements in MDI-treated patients are less than the overall 79 % reduction of insulin therapy previously observed in studies of type 2 diabetes patients, although this reduction was reported for a mean follow-up of 19.7 months [16]. This likely reflects the persisting absolute requirement for insulin therapy in patients with type 1 diabetes, as a result of immunological β-cell destruction and absolute insulin deficiency.

Previous case reports have described mixed effects of GB on type 1 diabetes. Czupryniak et al. found marked decreases in insulin requirements and HbA1c after GB in three patients who had poorly controlled type 1 diabetes preoperatively (mean HbA1c 10.4 %) [8, 10]. By contrast, a report from Mendez et al. in three patients showed no significant improvement in HbA1c, despite significant weight loss in three patients [9]. These differences could be due to the small sample size or differences in age, gender, time of subject assessment, and variable preoperative glycemic control. A recent paper assessed six patients undergoing a variety of bariatric surgical procedures, finding reductions in weight, insulin requirements, and HbA1c 1 year postoperatively; only two of these patients had undergone GB surgery [11].

In type 2 diabetes, GB improves insulin sensitivity, as defined by hyperinsulinemic euglycemic clamps [17], in parallel with reduced basal insulin levels and increased postprandial insulin secretion [6]. In type 1 diabetes, endogenous insulin levels are low to absent secondary to autoimmune beta cell destruction. Thus, the reduction in exogenous basal insulin requirement is likely related to resolution of obesity-induced insulin resistance. Additionally, reductions in total caloric intake and or altered proportions of macronutrients may also contribute to reduced insulin requirements. In our study, the reduction in basal insulin requirements did not correlate with the amount of weight loss, suggestive of a weight loss-independent mechanism. Moreover, GB yields marked effects on hepatic glucose metabolism as early as 1 week postoperatively, and although these data are derived from studies of individuals with type 2 diabetes [18], we hypothesize that similar effects could also be observed in severely obese patients with type 1 diabetes.

This study has several limitations. First, this is a small study of nine subjects, and the diagnosis of type 1 diabetes was defined clinically. However, five of nine subjects were diagnosed at <12 years of age, and laboratory confirmation was available in four subjects, including low or undetectable C-peptide levels and positive autoantibodies. Second, we assessed the impact on basal insulin levels in all subjects and meal bolus and total daily insulin doses as reported for MDI regimens, but we were unable to fully assess prandial doses in CSII-treated patients, since only insulin-to-carbohydrate ratios were reported. Third, our cohort was all female; while this is consistent with the preponderance of females undergoing bariatric surgery, these findings may not extend to men with type 1 diabetes who are treated with GB [7].

Despite these limitations, the present study is the largest to date which evaluates weight loss, glycemic control, and short-term changes in insulin requirements in patients with type 1 diabetes following GB. We specifically chose to follow subjects at short term (<20 weeks), to assess their insulin requirements before more major weight loss occurs and to provide guidance to clinicians who see these patients during the immediate postoperative and short-term follow-up visits after surgery. Further studies are clearly required to assess the durability of weight loss and improved glycemic control in patients with type 1 diabetes. Moreover, GB markedly alters patterns of early postprandial glycemia [4], contributing to a potential mismatch between nutrient absorption and timing of prandial insulin, with risk of subsequent hypoglycemia. Thus, it is often challenging to optimize prandial insulin therapy in post-GB patients. Additional studies will be required to determine optimal dose and timing of prandial insulin replacement in post-GB patients in the clinical setting.

In conclusion, we demonstrate that GB decreases BMI, HbA1c, and insulin requirements in severely obese women with type 1 diabetes. Consistent with the absolute requirement for insulin in type 1 diabetes, none of the subjects was able to discontinue insulin. Continuing an insulin regimen with post-GB patients with type 1 diabetes is critical in order to prevent onset of diabetic ketoacidosis, and frequent postoperative follow-up for adjustment of prandial insulin requirements is absolutely required. Longitudinal assessment will be important to evaluate long-term metabolic changes in our study population. Future larger and prospective studies will be needed to confirm these findings, assess the underlying metabolic changes, and evaluate the impact of GB on long-term morbidity and mortality in type 1 diabetes.
